# Measuring cannabis quantities in online surveys: A rapid review and proposals for ways forward

**DOI:** 10.1002/mpr.1971

**Published:** 2023-04-23

**Authors:** Jakob Manthey, Maria Teresa Pons‐Cabrera, Moritz Rosenkranz, Hugo Lopez‐Pelayo

**Affiliations:** ^1^ Department of Psychiatry and Psychotherapy Center for Interdisciplinary Addiction Research (ZIS) University Medical Center Hamburg‐Eppendorf (UKE) Hamburg Germany; ^2^ Department of Psychiatry Medical Faculty University of Leipzig Leipzig Germany; ^3^ Grup de Recerca en Addicions Clínic Institut d'Investigacions Biomèdiques August Pi i Sunyer (IDIBAPS) Unitat de Conductes Addictives Servei de Psiquiatria Psicologia (ICN) Hospital Clínic de Barcelona Barcelona Spain; ^4^ Red de Investigación en Atención Primaria de Adicciones (RIAPAd) Barcelona Spain

**Keywords:** cannabis, item, quantity, survey, visual aid

## Abstract

**Objectives:**

Cannabis use quantities are relevant for determining cannabis‐related harms. This research aims to provide an overview of the available methods to assess quantities through self‐report.

**Methods:**

A rapid review of various strategies to collect information on cannabis use quantities through self‐report. Two independent literature searches resulted in *n* = 38 studies included for review.

**Results:**

A total of *n* = 14 studies employed methods for collecting cannabis use quantities that are not suitable for online surveys (e.g., rolling a fake joint). Of the remaining *n* = 24 studies with items that are suitable for online surveys, the quantity assessment was performed in three different ways. The data collection was either carried out by asking (a) for the total number of joints (i.e., crude joint method), (b) for the total weight (i.e., crude weight method), or (c) for specific products separately, for example, for the amount of flower and resin (i.e., product‐specific method). In only *n* = 8 studies, cannabis use quantities were ascertained by providing visual aids (e.g., illustration of various amounts of flower).

**Conclusions:**

The crude joint method and the product‐specific method are the two most promising methods to collect information on cannabis use quantities. Using visual aids may potentially improve the accuracy of those methods.

## 1 INTRODUCTION

### 1.1 Background

In 2019, 3.9% of Europeans consumed cannabis in the last month, but only 0.5% met criteria for diagnosis of cannabis use disorder (Manthey et al., 2021). Other cannabis‐related harms beyond cannabis use disorder (e.g., respiratory symptoms, psychosis, motor‐vehicle accidents) are associated with regular use or risky use (Campeny et al., 2020), but definitions of risky use are not well‐established. Currently, our understanding of cannabis‐related harms and risky use is hampered by methodological imprecision when it comes to assessing variations in cannabis exposure. A wealth of empirical studies has shown that—in addition to frequency—the amounts used (on use days) and the concentration of tetrahydrocannabinol (THC) in used products are key to understand the risks associated with cannabis use (for quantity, see e.g (Asbridge et al., 2014; Callaghan et al., 2020); for THC, see for example (Di Forti et al., 2019)).

For a complete assessment of cannabis exposure in survey research, one could for example, use the product of (a) frequency of use day, (b) the number of joints on use days, and (c) the average amount of cannabis put into each joint (see e.g. (Korf et al., 2007)).

For (a) frequency of use—operationalized as the number of use days in a given period—appropriate items in several short questionnaires (e.g., CUDIT‐R (Adamson et al., 2010), ASSIST (WHO Assist Working Group, 2002)) are available. Moreover, frequency of use is recommended as one of three core items to screen for cannabis use (Lorenzetti et al., 2022).

For (b) and (c)—the quantity of cannabis used, there exists no validated, widely accepted tool. This is possibly due to this task being much more complex than assessing alcohol or tobacco use quantities, which are either standardized (tobacco: cigarettes or packs) or can be standardized by conversion factors (alcohol: standard drinks). For cannabis, quantitative assessment would optimally consider a range of factors that determine the actual exposure of THC, the main psychoactive compound of cannabis, such as product form (e.g., flower vs. resin vs. concentrate) and route of administration (eating vs. smoking vs. inhaling). The relevance of these factors, however, differs across regions depending on the predominant forms available (e.g., resin is the predominant use form in Morocco and Lebanon, while flower traditionally dominated the North American market) and cannabis culture (e.g., mixing and smoking with tobacco is much more common in European than North American countries (Hindocha et al., 2016)). Lastly, the dosage of the same product used in the same way (e.g., amount of flower per joint) may also differ across countries.

### 1.2 How are cannabis quantities currently assessed?

In a 2015 review on cannabis use questionnaires (Lopez‐Pelayo et al., 2015), the timeline follow‐back questionnaire was the only questionnaire identified to measure cannabis use quantities by asking users to specify the number of joints per day (Norberg, Mackenzie, & Copeland, 2012). One other validated survey is known to the authors that measures the average amount of cannabis flower used per typical session, day or week using a visual aid that depicts various amounts of flower (Cuttler & Spradlin, 2017). Further, several studies are known to the authors that have assessed cannabis quantities using either very short and simple (Zeisser et al., 2012) or long and comprehensive sets of items (see e.g. (Callaghan et al., 2019)).

It appears that numerous approaches to collect information on cannabis use quantities exist. However, the large variations in questionnaires in terms of length and wealth of information make it challenging for researchers to choose an appropriate method for measuring use quantities in (online) survey research as well as comparing results of different studies on cannabis‐related harms. With this contribution, we provide an overview of existing approaches and propose how quantitative assessments can be included in future surveys on cannabis users.

## 2 METHODS

Adhering to the Cochrane definition of a rapid review (The Cochrane Collaboration, 2023), this contribution aimed to provide a comprehensive, yet perhaps not complete, synthesis of the available evidence to identify ways forward for facilitating the assessment of cannabis quantities in online surveys. As a previous systematic review hardly found any instruments with validated cannabis quantity items (Lopez‐Pelayo et al., 2015), we aimed to summarize the methods currently employed by researchers.

For this purpose, we first performed a literature review of empirical studies that collected information on cannabis use quantities and THC exposure via standardization of means like standard joints. References on this topic were identified by searching PubMed and Web of Science databases (initial search: June 2021; updated: November 2022). The search terms included cannabis, marijuana, hashish, standardization, quantity, grams, standard unit, and joint unit (searched in “all fields” in PubMed; searched in “topic” in Web of Science) and yielded a total of *n* = 1661 studies, out of which *n* = 28 were finally included. As there were other studies known to the authors, including one validated instrument with a visual aid (Cuttler & Spradlin, 2017), an additional rapid search on PubMed was conducted on October 12, 2022 to ensure that studies using visual aids in collecting cannabis amounts are sufficiently covered in our review (search terms in “all fields”—default in PubMed: “(cannabis OR marijuana) and (quantity or amount) and (visual or prompt)”). That second search resulted in *n* = 46 studies of which an additional *n* = 4 studies were included. Lastly, experts in the field were contacted to ensure that we did not miss out on any relevant studies, but no additional study was included. Thus, a total of *n* = 38 studies were identified from various sources: search 1 (*n* = 28 studies), search 2 (*n* = 4 studies), a priori known to authors (*n* = 3 studies), screening of reference lists (*n* = 3 studies).

From all identified studies, information on the cannabis quantity items were extracted and summarized. At the first step, studies not suitable for online surveys were identified by requiring personal interaction between interviewers and interviewees (e.g., by collecting a fake joint) or the methods were considered to be too complex or time consuming for most online surveys.

Those studies that were deemed suitable for implementation in online surveys were grouped by using of visual aids in the quantitative assessment. In addition to describing the cannabis quantity items, our literature review has also covered information on psychometric properties, that is, estimates of validity and reliability.

The terms used for different cannabis products vary largely across studies, perhaps dependent on region and study period. For this current review we use the following terms: flower (synonymous to marijuana, leaf, dried herb, bud), resin (synonymous to hashish, kief), concentrate (synonymous to hash oil, butane hash or honey oil, shatter, wax).

## 3 RESULTS

### 3.1 Overview

**FIGURE 1:**
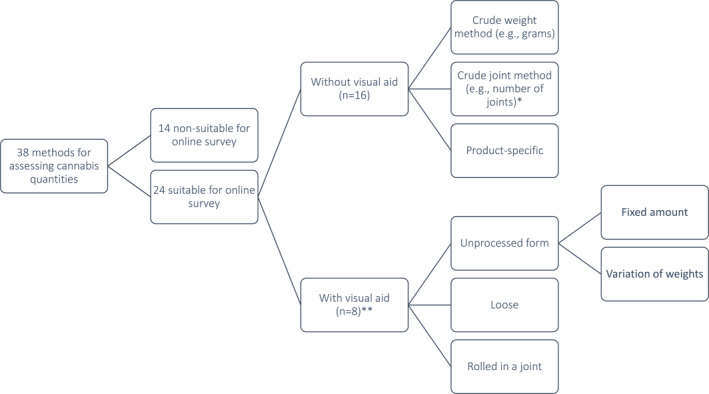
Summary of studies with assessment of cannabis quantities; * conversion of quantities between different products possible, for example, using standard THC units; ** with or without reference objects.

Overall, we identified a set of *n* = 38 studies in which information on cannabis use quantities were collected from users (see Figure 1).

For a sub‐set of *n* = 14 studies, the methods applied to collect information was not deemed suitable to be included in brief self‐report (online) surveys, as they involved rolling fake or actual joints (Casajuana Kögel et al., 2017; Hindocha et al., 2017; Mariani et al., 2011; Norberg, Mackenzie, & Copeland, 2012; Prince et al., 2018; Tomko et al., 2018), required trained interviewers (Connor et al., 2014), or were based on the lengthy timeline follow‐back (Buchowski et al., 2011; Copeland et al., 2017; Flanagan et al., 2020; Hjorthoj et al., 2013; Hjorthoj et al., 2012; Mariani et al., 2011; Norberg, Mackenzie, & Copeland, 2012; Norberg, Wright, et al., 2012; Savulich et al., 2021; Tomko et al., 2018) questionnaire. The remaining *n* = 24 studies collected information on cannabis use quantities using one or multiple questions with or without support of a visual aid to improve the accuracy of quantity estimations.

### 3.2 Studies without visual aids

Questions without visual aids were employed in *n* = 16 studies collecting information from cannabis users in Canada (Asbridge et al., 2014; Callaghan et al., 2019; Rotermann, 2019; Sikorski et al., 2021; Zeisser et al., 2012), Spain (Casajuana et al., 2016, 2018; Madero et al., 2020), USA (Callaghan et al., 2020; Caulkins et al., 2020; Chen et al., 2022; Lennox et al., 2006; Prince et al., 2020; Ridgeway & Kilmer, 2016; Trull et al., 2022) or USA/Australia (Bonn‐Miller et al., 2016) (see Table 1). In most studies, the cannabis quantities were collected for an average use day but some studies also referred to the peak amounts used (Lennox et al., 2006) or to light and heavy use days (Caulkins et al., 2020).

**TABLE 1:** Studies not using visual aids to assess cannabis use quantities.

Type of assessment	Study	Country	Item content	Response options	Dosing equivalents
Crude joint	Callaghan et al. (2020)	USA	Reporting the “number of joints usually smoked per day in last 12 months”	Not specified	None provided
Crude joint	Zeisser et al. (2012)	Canada	“During the past 30 days, on those days when you used marijuana, cannabis, or hashish, roughly how many joints did you usually have?”	1 = less than one joint a day; 2 = exactly one joint a day; 3 = more than one and up to three joints a day; 4 = 3.5 to 8 joints a day; 5 = 9 or more joints a day	1 joint = 0.5 g cannabis, 5 bong or pipe hits, or 10 puffs
Crude joint	Asbridge et al. (2014)	Canada	“On a typical day when you use cannabis, roughly how many joints do you usually have?”	Not specified	1 joint = 10 puffs, 5 bong or pipe hits, or 0.5 g
Crude joint	Casajuana et al. (2016)	Spain	Reporting the mean number of joints smoked per consumption day in the previous 30 days	Not specified	None provided
Crude joint	Casajuana et al. (2018)	Spain	Reporting the mean number of joints smoked per consumption day in the previous 30 days	Not specified	1 joint = 0.25 g of cannabis = 7 mg of THC
Crude joint	Chen et al. (2022)	USA	Reporting the number of joints	Not specified	None provided
Crude joint	Lennox et al. (2006)	USA	Reporting the peak amount of cannabis consumed during the past 90 days	Not specified	1 ounce = 25–30 joints; 1 dime = 4–5 joints; 1 nickel = 2–3 joints; 1 blunt = 2–6 joints; 1 g = 1–2 joints; 1 bowl = 1 joint; 10 1‐hit pipes = 1 joint
Crude joint	Madero et al. (2020)	Spain	Reporting the number of joints in the past week	Not specified	1 joint = 0.25 g of cannabis = 7 mg of THC
Crude joint	Prince et al. (2020)	USA	Reporting the number of joints per use episode	Not specified	1 joint = 0.5 g of cannabis
Crude weight	Trull et al. (2022)	USA	Reporting how many grams they had used since the last survey	1 = 1/8 g to 10 = over 2g	None provided
Crude weight	Caulkins et al. (2020)	USA	“How many grams or fractions of a gram do you think you usually use on a typical/light/heavy use day?”	Freetext	None provided
Crude joint/weight	Ridgeway and Kilmer, (2016)	USA	Reporting of cannabis used in terms of grams or ounces (crude weight), but also in terms of the number of bags, blunts, or joints (crude joint)	Not specified	None provided
Crude joint/weight	Bonn‐Miller et al. (2016)	USA/Australia	A) ‘‘How much cannabis (grams) do you usually use per week?’’ (crude weight); B) ‘‘On a typical day when you use cannabis, on average, how many cones, bongs, or joints do you normally have?’’ (crude joint)	A) 1 = 1 g, 2 = 2 g, 3 = 3–5 g, 4 = 6–8 g, 5 = 9–12 g, 6 = more than 12 g; B) not specified	None provided
Product‐specific	Sikorski et al. (2021)	Canada	Reporting of cannabis used on a usual day, week, month, or past 12‐months for 6 different cannabis products (flower, hashish/resin, hash oil, concentrate, tincture, topicals)	Not specified	1 hit resin or hash oil = 0.1 g or 1/5 bowl: 1 hit concentrate = 0.04 g, 1 drop tincture = 2 mL, 1 palmful topical = 1 ounce
Product‐specific	Callaghan et al. (2019)	Canada	Users to report the amounts for 7 different cannabis products (flower, hashish/resin, liquid concentrates, oil/cartridges, solid concentrates, edibles, drinks)	Depending on product—weight (e.g., grams) or numbers of units (e.g., 1 cartridge)	1 joint = 0.5 g flower, 0.125 g resin, 0.096 g concentrate, 65.8 oil drops, 7 edibles
Product‐specific	Rotermann, (2019)	Canada	Users to report the amounts for 8 different cannabis products (flower, oil/cartridges, hashish/resin, liquid concentrates, solid concentrates, edibles, other liquids, other)	Depending on product—weight (e.g., grams) or numbers of units (e.g., 1 cartridge)	Not converted

The studies differed mainly in conducting a simple, crude or a more comprehensive, product‐specific quantitative assessment. Studies with crude methods captured either the total weight or the total number of joints: for the “crude weight method”, users were asked to estimate either the quantity (weight) of cannabis used per day (e.g. (Trull et al., 2022)), while for a “crude joint method”, users were asked to estimate the number of joints they smoked per day (e.g. (Asbridge et al., 2014; Callaghan et al., 2020)). In some of these studies employing a crude assessment, the respondents were given information on how to convert various consumption forms themselves using a priori or empirically defined consumption equivalents (e.g., 1 joint equals 10 puffs, 5 bong or pipe hits, or 0.5 g flower (Asbridge et al., 2014)). Other studies asked for the number of joints and converted the responses into THC values based on prior data (e.g., average THC in a standard joint was defined on a sample of joints collected from users in the same region prior to the study (Madero et al., 2020)).

A product‐specific, rather than a crude method, was performed by some studies. The “product‐specific method” aimed to account for variations in THC and dosing across products, while minimizing errors due to the conversion between products by the respondents themselves. The product‐specific method included separate questions for several cannabis products (e.g. (Callaghan et al., 2019)) or administration forms (e.g., joints vs. bong: (Zeisser et al., 2012)). In those studies, the resulting product‐specific quantities were then converted into a common weight measure, again by using consumption equivalents that consider differences in mode of administration and THC concentrations. In one study, the product‐specific quantities were not converted due to many missing values in products other than flower (Rotermann, 2019).

### 3.3 Studies with visual aids

We identified *n* = 8 studies which collected information on cannabis use quantities with the help of visual aids (see Table 2 for a summary). The respective studies were conducted in Canada (Goodman et al., 2019), the Netherlands (Korf et al., 2007; van der Pol et al., 2013), and in the USA (Bonar et al., 2017; Collins et al., 2014; Cuttler & Spradlin, 2017; Pedersen et al., 2021), in addition to one multi‐country survey including data from Bulgaria, Czech Republic, Italy, Netherlands, Portugal, Sweden, and England & Wales (van Laar, Frijns, Trautmann, & Lombi, 2013). For three studies, the visual aids were not included in the publications and could also not be sourced from the corresponding authors upon request (Bonar et al., 2017; Collins et al., 2014; Pedersen et al., 2021). From the remaining five studies, four distinct sets of visual aids were identified from Cuttler and colleagues (Cuttler & Spradlin, 2017), Goodman and colleagues (Goodman et al., 2019), Korf and van der Pol (Korf et al., 2007; van der Pol et al., 2013), as well as van Laar and colleagues (van Laar et al., 2013).

**TABLE 2:** Studies using visual aids to assess cannabis use quantities.

Type of assessment	Study	Country	Reference period	Cannabis products displayed in which form	Reference object(s)	Visual aid accessible from publication
Crude weight	Bonar et al. (2017)	USA	Daily consumption (not further specified)	0.5 g cannabis (product type not mentioned) in rolled joints and loose form	Unknown	No
Crude weight	Collins et al. (2014)	USA	Amount used per use session	Approximately 0.5 g cannabis in rolled joints	Unknown	No
Crude weight	Cuttler and Spradlin (2017)	USA	Amount used on typical session/day/week	0.125 g/0.25 g/0.5 g/0.75 g/1g flower in loose and unprocessed form, as well as in rolled joints	1 US Dollar note	Yes
Product‐specific	Goodman et al. (2019)	Canada	Amount used per usual day/week/month or in the past 12‐months	Flower: 0.125 g/0.25 g/0.5 g/0.75 g/1 g/3.5 g (1/8 ounce)/7.1 g (1/4 ounce)/14.2 g (1/2 ounce)/28.4 g (1 ounce) in unprocessed form; Resin: 1g in unprocessed form; Concentrate: 0.5 g/1g in unprocessed form; Vape liquids/tinctures/liquid concentrates: 3 mL/5 mL/10 mL/15 mL in vials	Bottle cap	Yes
Product‐specific	Korf et al. (2007)	Netherlands	Amount used per joint	0.05 g/0.1 g/0.2 g/0.3 g flower and resin in loose and unprocessed form	Ruler and average joint	Yes
Crude weight	Pedersen et al. (2021)	USA	Amount used per occasion	0.25–5g (options in between not reported) flower (exact form not reported)	Unknown	No
Product‐specific	van der Pol et al. (2013)	Netherlands	Amount used per joint	0.05 g/0.1 g/0.2 g/0.3 g flower and resin in loose and unprocessed form	Ruler and average joint	Yes
Product‐specific	van Laar et al. (2013)	Netherlands	Amount used per joint	0.05 g/0.1 g/0.2 g/0.3 g flower and resin in loose and unprocessed form	Ruler and credit card	Yes

In all studies with available information, the quantitative assessment involved presenting one or more cannabis products (mostly flower but also resin) to the users. The displayed cannabis products were either presented as one fixed amount (e.g., 0.5 g: (Bonar et al., 2017; Collins et al., 2014)) or as a variation of weights (e.g. (Cuttler & Spradlin, 2017; Goodman et al., 2019; Korf et al., 2007; van der Pol et al., 2013; van Laar et al., 2013)) from which users could choose. The amounts presented varied largely (e.g., for flower: from 0.05 g (Korf et al., 2007; van der Pol et al., 2013) to 28.4 g (Goodman et al., 2019)) and perhaps reflect the reference period for which quantities were estimated (e.g., usual use session vs. in the past 12‐months)

**FIGURE A1:**
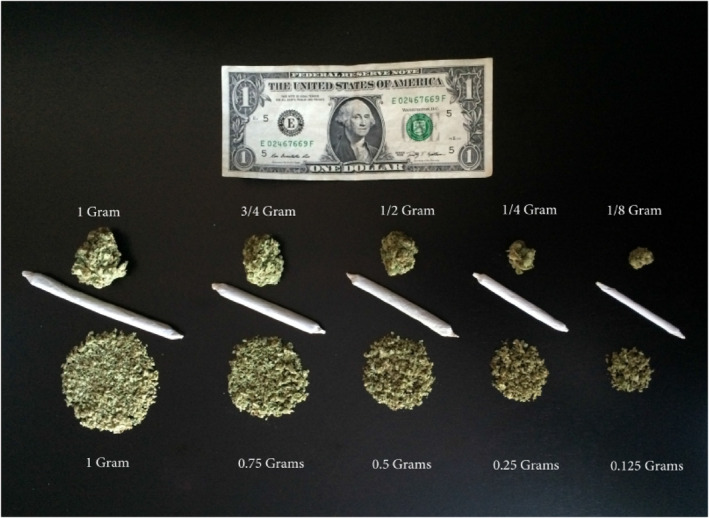
Visual aid to facilitate the assessment of cannabis quantities (crude weight method), taken from Cuttler et al. (Cuttler & Spradlin, 2017).

The cannabis products were either presented in unprocessed form (e.g., dried flower as bought from retailer), loose (i.e., ground for smoking for example in a joint), or rolled into a joint. When different amounts were presented, each form reflected the differing amounts by their size (e.g., larger joints for larger amounts). In one of the four sets of visual aids (Korf et al., 2007; van der Pol et al., 2013), various amounts were presented but the corresponding weight information was not included. Across the four sets of visual aids, different objects were used as reference objects to facilitate the understanding of the presented amounts: credit card (van Laar et al., 2013), ruler (Korf et al., 2007; van der Pol et al., 2013; van Laar et al., 2013), dollar note (Cuttler & Spradlin, 2017), bottle cap (Goodman et al., 2019), and an average joint (Korf et al., 2007; van der Pol et al., 2013). One example of a visual aid is presented as Appendix Figure A1.

### 3.4 Psychometric properties

The only comprehensive description of the psychometric properties of their quantity assessment was provided by Cuttler and Spradlin (2017). They showed that the cannabis quantity module loaded on a different factor than other survey modules, such as frequency or age of onset. Moreover, the three quantity items showed good internal consistency (*α* = 0.88). These items also showed some degree of convergent validity with other quantity measures (e.g., timeline follow‐back), some degree of predictive validity (e.g., with CAST or CUDIT) and discriminant validity with alcohol problems (i.e., no correlation with the AUDIT).

One study also assessed the validity of cannabis use quantities assessed with a visual aid by comparing the self‐reported amounts to actual, objectively measured amounts. With the visual aid, study participants estimated on average only half the actual amounts (0.13 g instead of 0.26 g) and underestimation was more pronounced among users with a cannabis dependence (van der Pol et al., 2013).

Further, five studies reported predictive validity for several measures of cannabis use problems. For example, using two or more joints per day was associated with higher ASSIST mean scores than using one joint per day (Asbridge et al., 2014). The validity of joints per day was also confirmed using the CAST as measure for CUD risk (Casajuana et al., 2018): on additional joint per day increased the odds of CUD risk by 44%.

One study also reported a correlation of the number of joints correlated with different types of individual cannabis problems (e.g., social, legal, and financial; urge to use; failure to do what expected)—but these associations mostly vanished when adjusting for cannabis use frequency (Zeisser et al., 2012). Prospective data also showed that higher amounts used at baseline predicted higher scores on the substance use scale and social risk index, but not to other scales describing illegal activities or emotional problems (Lennox et al., 2006). In a clinical sample, the number of joints was linked to higher psychopathology scores (psychotic, mania, anxiety, depressive symptoms) but this association was explained by other variables (e.g., diagnoses) in a multivariate model (Madero et al., 2020).

Lastly, one study correlated use quantities with cannabis use motives rather than problems. In adjusted models, quantities were associated with some but not all cannabis use motives (e.g, enhancement and coping; (Bonar et al., 2017)).

## 4 DISCUSSION

In this contribution, we reviewed *n* = 38 studies out of which *n* = 24 have used items assessing cannabis quantities that can be potentially included in future (online) surveys. Referring to the reported approaches already in use, we propose the following classification, starting with the shortest items:

### 4.1 Crude joint method

For the simplest quantitative assessment, users can be asked to indicate the number of joints on a typical use day. Aside from being a very brief measure, the advantage of this approach is that different products and distinct modes of administration can be assessed in terms of standard joints (for a proposed definition of standard joints, see (Freeman & Lorenzetti, 2020)). The prerequisite for this is to give users information on the definition of one standard joint (for example: 1 joint contains 0.5 g flower or is equivalent to 5 bong hits). While this supplementary information can possibly improve the accuracy, this measure still relies on respondents to make accurate reflections and calculations on the amounts and doses themselves, thereby, possibly biasing the estimates (e.g., by not factoring in heavy use days).

To further improve the crude joint method, a visual aid could be considered. In alcohol research, quantities in the widely used AUDIT‐C are commonly captured by asking for the average number of standard drinks per occasion. Very similar to the crude joint method, respondents are summing up the drinks across beverage categories themselves. In alcohol research, however, respondents' calculations are often facilitated by provision of a visual aid defining what one standard drink constitutes (see e.g. (Kuitunen‐Paul et al., 2017)). In this review, we have not identified a single study that used visual aids in the context of crude joint method, that is, visual aids that describe how various products or administration forms can be calculated into one joint. Adapting such visual aids in the crude joint method could be promising. For example, culturally‐adapted visual aids that describe how usual doses for various products can be converted into standard units (for proposals, see (Casajuana Kögel et al., 2017; Freeman & Lorenzetti, 2020)) could be an important step.

### 4.2 Crude weight method

Another simple way to collect information on cannabis quantities is asking users about the actual weight, rather than joints, used. The crude weight method does not make any assumptions on dosing (e.g., one joint contains 0.5 g flower), however, it relies on users' accurate estimations of the total weight used in a specified period, for example, in a usual week. To overcome this problem, several studies have used visual aids that are thought to improve the accuracy of respondents' weight estimates.

The key disadvantage of this method is that the total weight of cannabis used cannot consider variations in the product used, which can be illustrated in the following example. Person A uses mostly concentrates with THC levels of 90% and single doses of 0.05 g (i.e., 45 mg THC per dose), while person B uses mostly flower with THC levels of 20% and a single dose of 0.3 g (i.e., 60 mg THC per dose). If person A and person B both use 90 mg THC per day (2 doses for person A and 1.5 doses for person B), the crude joint method would be able to accurately capture the same levels of consumption. However, the crude weight method would result in 0.1 g cannabis for person A and 0.45 g for person B, thus, suggesting (large) differences in quantities.

### 4.3 Product‐specific method

The last and most elaborate quantitative assessment captures the quantity for a variety of products used in a specified period. Using the example from above, the two persons could indicate the total amount of each product they used on an average use day. Generally, this approach can be thought of as an extension of the crude joint method, but instead of letting users convert quantities across modes of administration and product types, this step is done by the researcher. Accordingly, information on weight (or other quantity indicators) are captured for each product (at least for flower and one other product, e.g., resin) separately. The product‐specific method can be supported by visual aids that presumably allow for a more accurate estimation of amounts across various products. In this review, four distinct sets of visual aids for product‐specific method have been identified. Differing in terms of reference objects used, amounts presented, as well as products and form presented, we observe a great heterogeneity in the visual aids and cannot recommend one over the other. However, one set of visual aids was evaluated in a feasibility study and items were rated overall feasible and/or changed based on the findings of the study (Goodman et al., 2019). Unfortunately, the revised items are not published.

While there are different methods for collecting information on cannabis use quantities available, there is no information on their relative accuracy. It can be assumed that the product‐specific method is more accurate than the crude methods and that visual aids generally improve the accuracy of data collection. While none of the included studies provide insights on these hypotheses, one study found that assessments using visual aids were underestimating the objectively measured dose by on average 50% (0.13 rather than 0.26 g cannabis per joint: (van der Pol et al., 2013)). Clearly, future research is required to develop accurate and culturally adapted measurement tools.

### 4.4 Limitations

While we have included a number of relevant studies that were identified through several literature searches, we do not claim to have covered all studies in this field. However, we believe that studies that were not considered in this rapid review are unlikely to change the proposed classification of methods to collect information on cannabis use quantities. Moreover, we focused on methods to collect information on cannabis use quantities in online surveys, which are inherently limited to biases arising from self‐report. Comparing self‐reported survey data with other data, for example, from biomarkers, may provide important information on the external validity of those assessments. Moreover, we restricted this rapid review on self‐report methods to collect data on cannabis use quantities and not explicitly on the psychoactive compounds, such as THC. The crude joint and product‐specific method methods can be used to account for variations between products or modes of administration, but within‐product variations may remain unknown. Further research will be required to determine the validity of those reviewed survey methods with respect to quantifying THC exposure (see e.g. (Trull et al., 2022)). Lastly, there is a lack of information on the psychometric properties for most of the presented items. The available data suggests that reliable and valid quantity assessments are feasible (see e.g., Cuttler and Spradlin (2017)) but there is also the risk of considerable under‐reporting (van der Pol et al., 2013).

## 5 CONCLUSION

There are several ways to collect information on cannabis use quantities in online surveys, with the crude joint method and the product‐specific method being the two most promising methods. The use of visual aids can potentially improve the accuracy of those assessments.

## CONFLICT OF INTEREST STATEMENT

Unrelated to the present work, Jakob Manthey has worked as consultant for and received honoraria from public health agencies; Jakob Manthey and Moritz Rosenkranz were involved in designing a study protocol for an experimental pilot study for licenced cannabis sales, funded by the federal state of Berlin; Maria Teresa Pons‐Cabrera has received financial support from Lundbeck, Pfyzer, and Esteve to attend meetings; Hugo Lopez‐Pelayo has received financial support from Lundbeck to attend meetings.

## Data Availability

All data generated or analysed during this literature review are included in this published article.
